# Variability of urinary albumin to creatinine ratio and eGFR are independently associated with eGFR slope in Japanese with type 2 diabetes: a three-year, single-center, retrospective cohort study

**DOI:** 10.1186/s12882-024-03699-4

**Published:** 2024-08-16

**Authors:** Takaaki Matsuda, Yoshinori Osaki, Kazushi Maruo, Erika Matsuda, Yasuhiro Suzuki, Hiroaki Suzuki, Bryan J. Mathis, Hitoshi Shimano, Masakazu Mizutani

**Affiliations:** 1 Department of Internal Medicine, Kozawa Eye Hospital and Diabetes Center, 246-6 Yoshizawa-cho, Mito, Ibaraki 310-0845 Japan; 2 Department of Endocrinology and Metabolism, Institute of Medicine, University of Tsukuba, 1-1-1 Tennodai, Tsukuba, Ibaraki 305-8575 Japan; 3 Tsukuba Clinical Research and Development Organization (T-CReDO), University of Tsukuba, Tsukuba, Ibaraki 305-8575 Japan; 4 Institute of Systems and Information Engineering, University of Tsukuba, Tsukuba, Ibaraki 305-8573 Japan; 5 Department of Food and Health Sciences, Faculty of Human Life Sciences, Jissen Women’s University, Hino, Tokyo, 191-8510 Japan; 6 Department of Cardiovascular Surgery, Institute of Medicine, University of Tsukuba, Tsukuba, Ibaraki 305-8575 Japan

**Keywords:** Diabetes mellitus, Type 2, Diabetic nephropathies, Seasonal variation

## Abstract

**Background:**

To evaluate the seasonal variability of urinary albumin to creatinine ratio (UACR) and eGFR and these effects on three-year eGFR slope in persons with type 2 diabetes (T2D).

**Methods:**

A total of 1135 persons with T2D were analyzed in this single-center, retrospective cohort study in Japan. The standard deviation (SD) of UACR (SD [UACR]) and SD of eGFR (SD [eGFR]) were calculated for each person’s 10-point data during the three years, and a multiple linear regression analysis was performed to evaluate associations with eGFR slope. A sensitivity analysis was performed in a group with no medication changes (*n* = 801).

**Results:**

UACR exhibited seasonal variability, being higher in winter and lower in spring, early summer, and autumn especially in the UACR ≥ 30 mg/g subgroup, while eGFR showed no seasonal variability. The eGFR slope was significantly associated with SD (eGFR) (regression coefficient -0.170 [95% CI -0.189–-0.151]) and SD (UACR) (0.000 [-0.001–0.000]). SGLT-2 inhibitors, baseline eGFR, and baseline systolic blood pressure (SBP) were also significantly associated. These associated factors, except baseline SBP, were still significant in the sensitivity analysis.

**Conclusions:**

The UACR showed clear seasonal variability. Moreover, SD (UACR) and SD (eGFR) were independently associated with a three-year eGFR slope in persons with T2D.

**Trial registration:**

This study was not registered for clinical trial registration because it was a retrospective observational study.

**Supplementary Information:**

The online version contains supplementary material available at 10.1186/s12882-024-03699-4.

## Background

Diabetic kidney disease (DKD) is a microvascular complication of diabetes mellitus and a major cause of end-stage renal disease [1]. Guidelines for chronic kidney disease (CKD) or DKD recommend that DKD be diagnosed based on urinary albumin excretion (UAE) and renal function as represented by estimated glomerular filtration rate (eGFR) [2–4]. Although UAE is closely associated with increased risks of mortality and cardiovascular disease [5, 6], the post-hoc analysis of Action in Diabetes and Vascular Disease: PreterAx and DiamicroN-MR Controlled Evaluation (ADVANCE) study showed that UAE and eGFR are independently associated with negative outcomes [7].

Clinical practitioners constantly assess risk factors associated with progressive renal dysfunction in individuals with diabetes [8]. The Clinical Practice Guideline for the Evaluation and Management of Chronic Kidney Disease thus reports several risk factors: baseline level of eGFR and albuminuria, age, sex, blood pressure, blood glucose, dyslipidemia, smoking, obesity, and history of cardiovascular disease [9]. The cumulative impact of these risk factors is used to predict eGFR deterioration in persons with type 2 diabetes (T2D) [10]. In addition, previous studies have already reported associations between DKD progression and variability of HbA1c [11, 12] and blood pressure [12, 13] readings in persons with T2D. In those studies, variability indices were associated with deterioration of renal function independently of mean values [12, 13].

Nevertheless, any associations between urinary albumin to creatinine ratio (UACR)/eGFR variability and changes in eGFR remain partially evaluated. While reports on eGFR exist from several studies [14, 15], full knowledge of eGFR variability with respect to slope and seasonal timing within T2D populations is poorly described. A post-hoc analysis of the ADVANCE trial reported that the highest eGFR variability group had a significantly higher risk of new-onset/worsening of diabetic nephropathy compared with the lowest eGFR variability group [14]. Additionally, a retrospective observational study targeting individuals with diabetes in CKD (stage 3–4) reported associations between eGFR variability and risks of dialysis or death [15]. However, a scarcity of reports evaluating associations between eGFR variability and changes in eGFR (represented by eGFR slope) in persons with T2D persists. Moreover, the seasonal variability of UACR in persons with T2D was reported as higher in winter and lower in summer (in T2D cases with microalbuminuria) in a single-cohort Japanese study [16]; no reports have since evaluated eGFR seasonal variability in persons with T2D.

The purpose of this three-year retrospective cohort study was therefore to extend current knowledge by examining seasonal variabilities of UACR and eGFR and the subsequent effect on eGFR changes in Japanese with T2D.

## Methods

### Study design and settings

This was a single-center, three-year retrospective cohort study performed at the outpatient department of the Kozawa Eye Hospital and Diabetes Center.

### Participants

We enrolled persons with T2D who visited the hospital from November 2018 to March 2019; the data collection period was three years (until April 2022). The exclusion criteria were as follows: i) diabetes other than T2D, ii) non-measurement of UACR during the study period, iii) diagnosed or initiated therapy within 6 months, iv) entry into a clinical trial for a DKD therapeutic agent, v) died during the study period, vi) comorbidity with another kidney disease, and vii) under 18 years old. We excluded individuals within six months of diabetes diagnosis or treatment because they were more likely to experience rapid improvement in glycemic control, which could affect renal function. We chose six months based on the fact that renal function as measured by eGFR stabilized around six months in clinical trials with SGLT2 inhibitors [17].

### Variables and data collection

We retrospectively collected the following data during the study period: body weight (BW), systolic blood pressure (SBP), diastolic blood pressure (DBP), UACR, HbA1c, serum creatinine, serum albumin, uric acid (UA), low-density lipoprotein cholesterol (LDL-C), high-density lipoprotein cholesterol (HDL-C), and triglyceride (TG) levels. Body mass index (BMI) was calculated by dividing BW (kg) by the square of height (m^2^).

Estimated glomerular filtration rate (eGFR) was calculated using a Chronic Kidney Disease Epidemiology Collaboration (CKD-EPI) equation [18, 19]. We used eGFR values based on the CKD-EPI multiplied by a Japanese correction factor (multiply by 0.813) [20].

The reference data acquisition points were January, May, and September 2019; January, May, and September 2020; February, June, and October 2021; and February 2022, when regular UACR measurements were conducted at our hospital, and the allowable range was two months before or after. In Japan, January/February, May, June, and September/October correspond to winter, spring, early summer, and autumn, respectively.

Participant medical and laboratory data were collected from electronic medical records; however, information on diabetic neuropathy was unlisted due to a lack of sufficient data. Cardiovascular diseases included stroke, ischemic heart disease, and peripheral arterial disease.

### Outcomes

The outcome of this study included the eGFR slope (mL/min/1.73 m^2^/year) over three years. The eGFR slope was calculated from the slope of a linear approximation of eGFR value and days based on ten eGFR data points from each participant. We evaluated three-year eGFR slopes by UACR category (UACR < 30 mg/g, UACR 30–300 mg/g, or UACR ≥ 300 mg/g) and eGFR category (eGFR ≥ 90 mL/min/1.73 m^2^, eGFR 60–90 mL/min/1.73 m^2^, or eGFR < 60 mL/min/1.73 m^2^) for subgroup analysis. Since few participants had an eGFR below 60 mL/min/1.73 m^2^, this category was not subdivided. Seasonal variability in UACR and eGFR was also evaluated using the ten-timepoint series data and compared to changes in UACR and eGFR from baseline at each data point.

### Methods of measurement

Blood pressure was measured once by an automatic sphygmomanometer or a nurse in the hospital. Body weight was measured using a scale with light clothes and without adjustment. We used spot urine samples to measure the UACR, which was performed three to six hours after urine collection using a turbidimetric immunoassay (Nittobo Medical, Tokyo, Japan). Blood samples were collected in the fasting or postprandial states. HbA1c was immediately measured in our hospital via high-performance liquid chromatography (HPLC) with an automated analyzer (Arkray, Kyoto, Japan). Serum levels of creatinine, albumin, UA, LDL-C, HDL-C, and TG were measured by automated analyzer (LABOSPECT 008, Hitachi High-Technologies Corp., Japan).

### Covariates

We collected baseline demographic variables for covariates as follows: age, sex, height, smoking (including ex-smokers), duration of diabetes, past medical history or comorbidity (hypertension, dyslipidemia, diabetic retinopathy, and/or cardiovascular disease), and therapeutic agents (glucose-lowering drugs, antihypertensive drugs, diuretics, antihyperlipidemic drugs, anti-hyperuricemia drugs, and/or antiplatelet drugs).

Furthermore, we collected medication statuses in January 2020 and February 2022 to identify drug changes. We defined those participants whose medications (sodium-glucose cotransporter-2 [SGLT-2] inhibitors, glucagon-like peptide-1 receptor agonists [GLP-1 RAs], renin-angiotensin system [RAS] inhibitors including angiotensin receptor blockers and angiotensin-converting enzyme inhibitors, calcium channel blockers, mineral corticoid receptor antagonists, loop diuretics, and/or thiazides) had not changed (*i.e.*, those not newly added or discontinued) at the January 2020 and February 2022 timepoints as the “non-medication-changes” group.

### Ethical approval

This study, which complied with the Declaration of Helsinki, was approved by the Kozawa Eye Hospital and Diabetes Center Ethics Review Board which belongs to Kozawa Eye Hospital and Diabetes Center (The date of approval was June 14, 2022, approval number KG2022-23).

### Statistical analysis

The baseline data of the study participants are represented based on their variable characteristics. Continuous variables are represented as means (standard deviations [SD]) or median (interquartile range) based on distribution. Categorical variables are presented as numbers (percentages). We included participants who discontinued therapy or were introduced to other doctors to maintain cohort size and avoid incomplete case analysis. In terms of missing values, we performed the multiple imputation with the fully conditional specification method [21] 100 times. The imputation model included baseline demographic variables and clinical time-series data. Since the distribution of UACR was heavily skewed, the data were converted using logarithmic transformation at the time of statistical analysis and log-transformed variables for statistical analysis are listed.

The seasonal variability analysis was performed using paired t-tests at each data point from baseline. We calculated the SDs of UACR, eGFR, HbA1c, and SBP for each person using data from 10-time points over three years as variables representing individual’s variability and represented them as SD (UACR), SD (eGFR), SD (HbA1c), and SD (SBP). We used these variables as dependent variables in multivariable regression analyses.

A multivariable regression analysis was performed to evaluate associations between UACR and eGFR variability, as well as the three-year changes in eGFR slopes. We adopted the eGFR slope as a dependent variable and adjusted it with prespecified variables; Model 1 was unadjusted while Model 2 was adjusted with age, sex, duration of diabetes, smoking, past medical history or comorbidity (hypertension, dyslipidemia, diabetic retinopathy, and/or cardiovascular disease), therapeutic agents (SGLT-2 inhibitors, GLP-1 RAs, RAS inhibitors, calcium channel blockers, mineral corticoid receptor antagonists, loop diuretics, and/or thiazides), baseline clinical data (UACR [log-transformed], BMI, SBP, eGFR, and HbA1c), and SD (UACR), SD (eGFR), SD (HbA1c), and SD (SBP). We selected these variables, except SD (UACR) and SD (eGFR), based on previous studies evaluating associations between exposure and kidney function [9, 11–13, 22–26]. Moreover, we also conducted a multivariable regression analysis for sensitivity in the non-medication-changes group during the study period to minimize the impact of medication changes.

R software ver. 4.3.1 (R Core Team, Vienna, Austria) was used for all data analyses. Significance level for all statistical tests was set at 0.05.

## Results

### Study participant flow

**Fig. 1:**
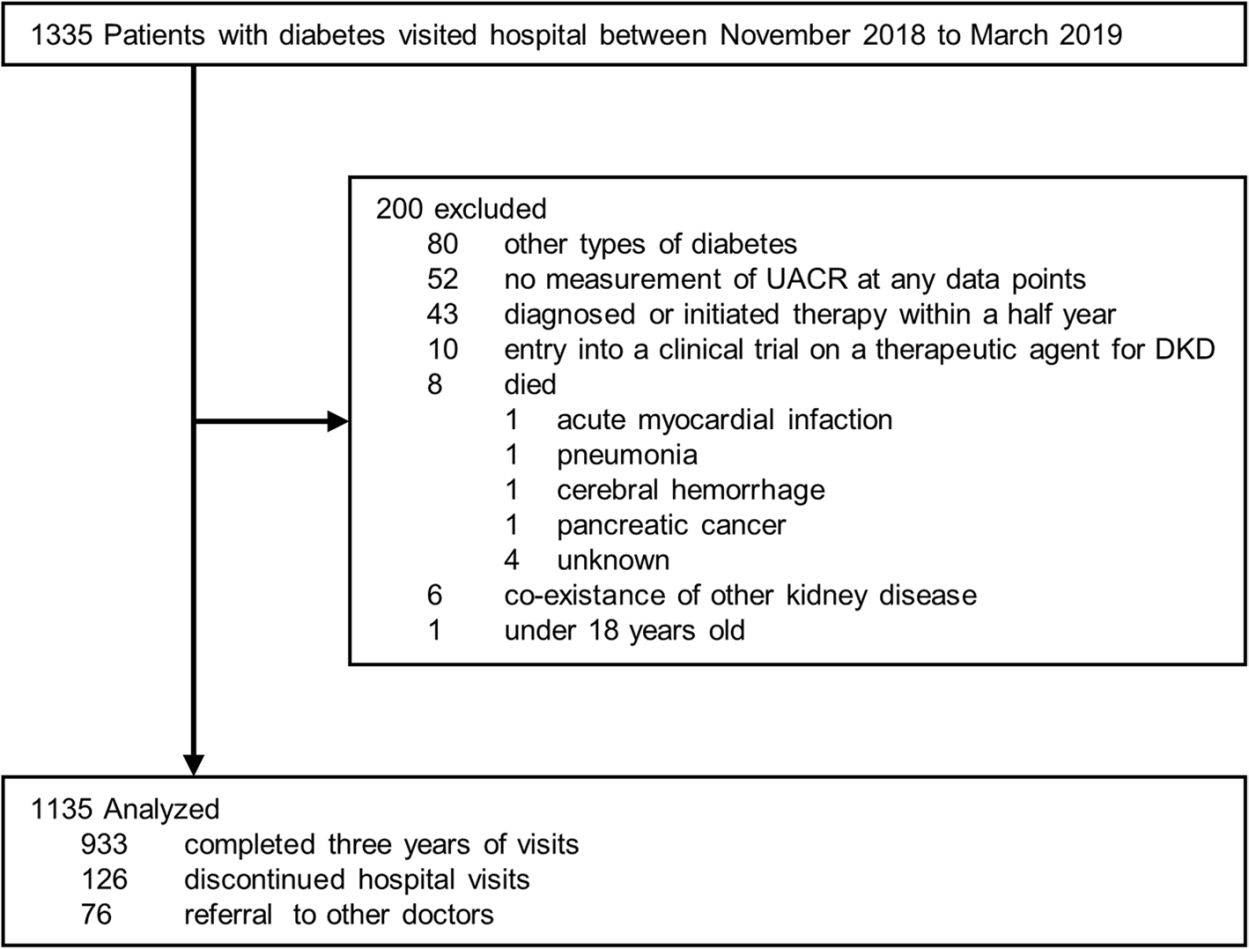
Flowchart of study participants. *UACR* urinary albumin to creatinine ratio, *DKD* diabetic kidney disease

A total of 1335 patients with diabetes visited our hospital between November 2018 to March 2019. We excluded 200 cases (other types of diabetes [*n* = 80], non-measurement of UACR during the study period [*n* = 52], diagnosed or initiated therapy within a half year [*n* = 43], entry into a clinical trial on a therapeutic agent for DKD [*n* = 10], died during the study period [*n* = 8], comorbidity of other kidney disease [*n* = 6], or under 18 years old [*n* = 1]). After exclusion, 1135 patients were included in the final analysis (Fig. 1) and 801 patients were eligible for the sensitivity analysis (non-medication-changes group).

### Baseline characteristics of study participants

Table 1 shows the baseline characteristics of the study participants. The mean age was 66 (11) years old and 39% were female. The mean duration of diabetes was 13 (9) years. The mean SBP and DBP were 136 (16) mmHg and 77 (11) mmHg, respectively. The mean HbA1c level was 7.3 (1.1) % and 56 (12) mmol/mol. The proportions of participants with hypertension and dyslipidemia were 67% and 53%. The proportions of patients were categorized by baseline UACR as follows: 51.1% for UACR < 30 mg/g, 38.3% for UACR 30–300 mg/g, and 10.6% for UACR ≥ 300 mg/g. The use proportions of SGLT-2 inhibitors, GLP-1 RAs, RAS inhibitors, loop diuretics, or thiazides was 21%, 3%, 47%, 2%, and 4%, respectively.

**Table 1:** Baseline characteristics of study participants

Variables	All Study Participants Group (*n* = 1135)	Non-Medication-Changes Group (*n* = 801)	Medication-Changes Group (*n* = 334)	P for Comparing Groups without and with Medication Change
Age (years)	66 (11)	66 (11)	64 (12)	0.001
Female	437 (38.5)	315 (39.3)	122 (36.5)	0.414
Duration of Diabetes (y)	13 (9)	14 (9)	13 (9)	0.123
Smoking	479 (42.2)	327 (40.8)	152 (45.5)	0.109
BMI (kg/m^2^)	25.2 (4.2)	24.8 (4.0)	26.2 (4.4)	< 0.001
SBP (mmHg)	136 (16)	135 (16)	137 (17)	0.045
DBP (mmHg)	77 (11)	76 (11)	79 (12)	< 0.001
HbA1c (%)	7.3 (1.2)	7.2 (1.1)	7.6 (1.3)	< 0.001
HbA1c (mmol/mol)	56 (13)	55 (12)	59 (14)	< 0.001
UACR (mg/g)	29 (12–92)	26 (11–85)	36 (15–121)	0.049
eGFR (mL/min/1.73 m^2^)	74 (15)	74 (14)	74 (16)	0.977
Baseline eGFR Category				
≥ 90	104 (9.16)	64 (7.99)	40 (11.98)	0.102
60–90	852 (75.07)	412 (76.40)	240 (71.86)	
< 60	179 (15.77)	125 (15.61)	54 (16.17)	
Serum Albumin (g/dL)	4.3 (0.2)	4.3 (0.2)	4.3 (0.2)	0.544
Uric Acid (mg/dL)	5.4 (1.4)	5.2 (1.2)	5.4 (1.4)	0.125
LDL-C (mg/dL)	115 (29)	113 (28)	115 (31)	0.187
HDL-C (mg/dL)	53 (16)	57 (17)	53 (14)	< 0.001
TG (mg/dL)	189 (176)	160 (116)	189 (176)	0.001
Hypertension	763 (67.2)	510 (63.7)	253 (75.8)	< 0.001
Dyslipidemia	596 (52.5)	414 (51.7)	182 (54.5)	0.425
Diabetic Complications				
Retinopathy	495 (43.6)	334 (41.7)	164 (49.1)	0.041
Nephropathy				
UACR < 30 mg/g	580 (51.1)	428 (53.4)	152 (45.5)	0.195
UACR 30–300 mg/g	435 (38.3)	294 (36.7)	141 (42.2)	
UACR ≥ 300 mg/g	120 (10.6)	79 (9.9)	41 (12.3)	
Cardiovascular Disease	175 (15)	131 (16)	44 (13)	0.224
Therapeutic Drugs				
SGLT-2 Inhibitors	239 (21.1)	146 (18.2)	93 (27.8)	< 0.001
GLP-1 RAs	33 (2.9)	15 (1.9)	18 (5.4)	0.003
Metformin	540 (47.6)	353 (44.1)	187 (56.0)	< 0.001
DPP-4 Inhibitors	744 (65.6)	509 (63.5)	235 (70.4)	0.033
Sulfonylureas	177 (15.6)	124 (15.5)	53 (15.9)	0.941
Glinides	147 (13.0)	111 (13.9)	36 (10.8)	0.190
Thiazolidines	48 (4.2)	33 (4.1)	15 (4.5)	0.903
α-glucosidase Inhibitors	182 (16.0)	129 (16.1)	53 (15.9)	0.992
Insulin	152 (13.4)	101 (12.6)	51 (15.3)	0.270
RAS Inhibitors	536 (47.2)	340 (42.5)	196 (58.7)	< 0.001
Calcium Channel Blockers	402 (35.4)	263 (32.8)	139 (41.6)	0.006
Mineralcorticoid Receptor Antagonists	9 (0.8)	6 (0.8)	3 (0.9)	1.000
Loop Diuretics	17 (1.5)	10 (1.3)	7 (2.1)	0.422
Thiazide	43 (3.8)	20 (2.5)	23 (6.9)	0.001
α-blockers	5 (0.4)	3 (0.4)	2 (0.6)	0.978
β-blockers	37 (3.3)	28 (3.5)	9 (2.7)	0.611
Statins	321 (28.3)	224 (28.0)	97 (29.0)	0.768
Ezetimibe	38 (3.4)	29 (3.6)	9 (2.7)	0.542
Fibrates	52 (4.6)	40 (5.0)	12 (3.6)	0.383
EPA/DHA	36 (3.2)	26 (3.3)	10 (3.0)	0.972
Antihyperuricemic Drugs	75 (6.6)	48 (6.0)	27 (8.1)	0.245
Antiplatelet Drugs	132 (11.6)	102 (12.7)	30 (9.0)	0.090
Anticoagulant Drugs	14 (1.2)	7 (0.9)	7 (2.1)	0.160

Data are expressed as the mean (SD), median (interquartile range), and %. Statistical comparison of the background factors in the non-medication-changes and medication-changes groups is shown by *P*-values

*BMI* Body mass index, *SBP* Systolic blood pressure, *DBP* Diastolic blood pressure, *UACR* Urinary albumin to creatinine ratio, *eGFR* estimated glomerular filtration rate, *LDL-C* Low-density lipoprotein cholesterol, *HDL-C* High-density lipoprotein cholesterol, *TG* Triglycerides, *SGLT-2* Sodium-glucose cotransporter-2, *GLP-1RAs* Glucagon-like peptide-1 receptor agonists, *DPP-4* Dipeptidyl peptidase-4, *RAS* Renin-angiotensin system, *EPA/DHA* Eicosapentaenoic acid/docosahexaenoic acid, *SD* Standard deviation

We compared baseline characteristics between the non-medication-changes and medication-changes groups, identifying significant differences in the following variables: age, BMI, SBP, DBP, HbA1c, UACR, HDL-C, TG, past medical history or comorbidity (hypertension and retinopathy), and medication use (SGLT-2 inhibitors, GLP-1 RAs, metformin, dipeptidyl peptidase-4 [DPP-4] inhibitors, RAS inhibitors, calcium channel blockers, and/or thiazide).

### Seasonal variability of UACR and eGFR

**Fig. 2:**
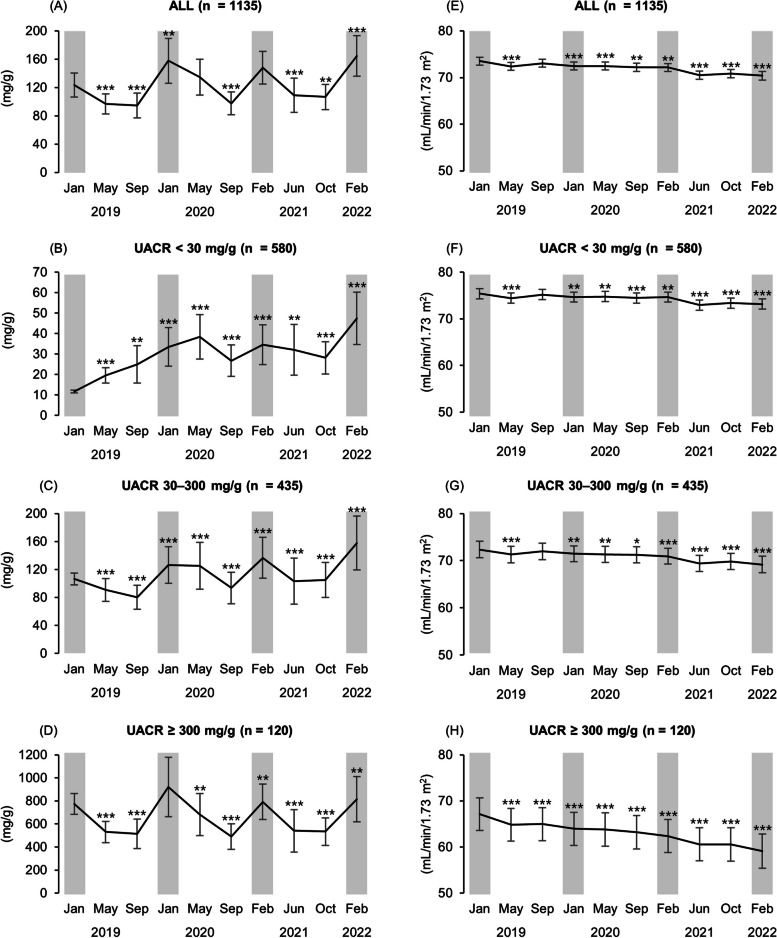
Seasonal variability of UACR and eGFR. Seasonal variability of UACR in all groups. **A** UACR < 30 mg/g (**B**) UACR 30–300 mg/g (**C**) UACR ≥ 300 mg/g. **D** Seasonal variability of eGFR in all groups. **E** UACR < 30 mg/g (**F**) UACR 30–300 mg/g (**G**) UACR ≥ 300 mg/g (**H**). The vertical axis in (**A**), (**B**), (**C**), and (**D**) represents the UACR value (mg/g) while the vertical axis in (**E**), (**F**), (**G**), and (**H**) represents the eGFR value (mL/min/1.73 m^2^). The horizontal axis represents a time series from January 2019 to February 2022. Error bars in the graphs represent 95% confidence intervals. The gray zone indicates winter months. ^*^P < 0.05, ^**^P < 0.01, and ^***^P < 0.001 vs. baseline. *UACR* urinary albumin to creatinine ratio, *eGFR* estimated glomerular filtration rate

Seasonal variability in UACR was observed, with values significantly lower in spring, early summer, and autumn compared to winter (Fig. 2). UACR seasonal variability was higher in the UACR ≥ 30 mg/g groups than the UACR < 30 mg/g group. Conversely, eGFR did not demonstrate seasonal variability in any group. In the subgroup analysis, compared with the baseline, the eGFR at three years deteriorated as the UACR category stage worsened.

### Associations between three-year eGFR slope and variability of UACR, eGFR, SBP, and HbA1c

A multiple linear regression analysis showed that the three-year eGFR slope was significantly associated with SD (UACR), SD (eGFR), SGLT-2 inhibitors, and baseline SBP and eGFR in all study groups (Table 2).

**Table 2:** Associations between a three-year change in eGFR slope and variability of UACR, SBP, eGFR, and HbA1c (multiple linear regression analysis)

Independent Variables	All				UACR Category (mg/g)									
					< 30				30–300				300 ≤	
	(*n* = 1135)				(*n* = 580)				(*n* = 435)				(*n* = 120)	
	Model 1		Model 2		Model 1		Model 2		Model 1		Model 2		Model 1	
	β (95% CI)	P	β (95% CI)	P	β (95% CI)	P	β (95% CI)	P	β (95% CI)	P	β (95% CI)	P	β (95% CI)	P
SD (UACR)	-0.001 (-0.001–-0.001)	<0.001	0.000 (-0.001–0.000)	<0.001	-0.001 (-0.002–0.000)	0.089	0.000 (-0.001–0.000)	0.272	-0.001 (-0.002–0.000)	0.003	0.000 (-0.001–0.000)	0.127	-0.001 (-0.001–0.000)	0.015
SD (eGFR)	-0.157 (-0.177–-0.138)	<0.001	-0.170 (-0.189–-0.151)	<0.001	-0.110 (-0.142–-0.077)	<0.001	-0.147 (-0.181–-0.113)	<0.001	-0.151 (-0.192–-0.110)	<0.001	-0.169 (-0.214–-0.124)	<0.001	-0.256 (-0.317–-0.194)	<0.001
SD (HbA1c)	-0.168 (-0.299–-0.038)	0.012	0.124 (-0.002–0.250)	0.053	-0.018 (-0.171–0.134)	0.816	0.102 (-0.056–0.260)	0.205	-0.205 (-0.466–0.056)	0.123	0.047 (-0.208–0.303)	0.717	-0.361 (-0.911–0.188)	0.195
SD (SBP)	-0.019 (-0.030–-0.007)	0.002	0.005 (-0.006–0.015)	0.392	-0.008 (-0.021–0.005)	0.228	0.001 (-0.012–0.013)	0.888	-0.010 (-0.031–0.011)	0.355	0.011 (-0.009–0.031)	0.287	-0.045 (-0.090–0.000)	0.049
SGLT-2 Inhibitors	0.181 (0.079–0.284)	0.001	0.160 (0.065–0.254)	0.001	0.159 (0.039–0.279)	0.009	0.144 (0.019–0.268)	0.024	0.223 (0.039–0.407)	0.018	0.175 (0.003–0.347)	0.046	0.231 (-0.209–0.671)	0.301
RAS Inhibitors	-0.069 (-0.153–0.014)	0.102	-0.034 (-0.117–0.048)	0.412	-0.086 (-0.181–0.010)	0.079	-0.069 (-0.173–0.034)	0.189	0.016 (-0.144–0.177)	0.842	-0.060 (-0.221–0.101)	0.466	0.138 (-0.257–0.533)	0.490
UACR	-0.088 (-0.117–-0.060)	<0.001	-0.029 (-0.058–0.001)	0.059	-0.049 (-0.122–0.024)	0.190	-0.040 (-0.107–0.028)	0.246	-0.038 (-0.166–0.089)	0.555	0.011 (-0.102–0.125)	0.846	-0.638 (-1.000–-0.275)	0.001
BMI	0.005 (-0.006–0.015)	0.378	0.002 (-0.008–0.011)	0.701	0.004 (-0.007–0.015)	0.465	0.003 (-0.009–0.014)	0.659	0.010 (-0.011–0.031)	0.361	0.001 (-0.019–0.022)	0.891	0.006 (-0.038–0.051)	0.777
SBP	-0.005 (-0.008–-0.003)	<0.001	-0.003 (-0.005–0.000)	0.031	-0.003 (-0.006–0.000)	0.050	-0.002 (-0.005–0.001)	0.136	-0.005 (-0.010–0.000)	0.070	-0.002 (-0.007–0.002)	0.338	-0.005 (-0.015–0.006)	0.412
eGFR	-0.007 (-0.010–-0.004)	<0.001	-0.021 (-0.024–-0.017)	<0.001	-0.006 (-0.010–-0.002)	0.002	-0.022 (-0.028–-0.016)	<0.001	-0.011 (-0.016–-0.005)	<0.001	-0.022 (-0.029–-0.015)	<0.001	-0.010 (-0.020–0.000)	0.049
HbA1c	-0.036 (-0.073–0.002)	0.062	-0.022 (-0.059–0.015)	0.249	0.004 (-0.043–0.050)	0.880	0.014 (-0.036–0.064)	0.582	-0.042 (-0.112–0.028)	0.236	-0.033 (-0.106–0.040)	0.377	-0.084 (-0.218–0.051)	0.221

Independent Variables			eGFR Category (mL/min/1.73 m^2^)											
			90 ≤				60–90				< 60			
			(*n* = 104)				(*n* = 852)				*(n* = 179)			
	Model 2		Model 1		Model 2		Model 1		Model 2		Model 1		Model 2	
	β (95% CI)	P	β (95% CI)	P	β (95% CI)	P	β (95% CI)	P	β (95% CI)	P	β (95% CI)	P	β (95% CI)	P
SD (UACR)	0.000 (-0.001–0.000)	0.805	-0.001 (-0.002–0.000)	0.055	0.000 (-0.002–0.001)	0.759	-0.001 (-0.002–-0.001)	<0.001	0.000 (-0.001–0.000)	<0.001	-0.001 (-0.001–0.000)	0.013	0.000 (-0.001–0.000)	0.206
SD (eGFR)	-0.246 (-0.321–-0.172)	<0.001	-0.146 (-0.205–-0.088)	<0.001	-0.134 (-0.210–-0.058)	0.001	-0.197 (-0.214–-0.179)	<0.001	-0.211 (-0.230–-0.192)	<0.001	-0.082 (-0.159–-0.005)	0.037	-0.057 (-0.136–0.023)	0.159
SD (HbA1c)	0.424 (-0.083–0.932)	0.100	-0.297 (-0.586–-0.009)	0.043	-0.206 (-0.571–0.159)	0.263	-0.110 (-0.261–0.041)	0.153	0.149 (0.026–0.272)	0.018	-0.127 (-0.615–0.360)	0.606	0.288 (-0.299–0.875)	0.333
SD (SBP)	0.002 (-0.036–0.041)	0.900	-0.040 (-0.077–-0.004)	0.030	-0.013 (-0.050–0.024)	0.484	-0.020 (-0.033–-0.007)	0.002	0.009 (-0.001–0.019)	0.063	-0.028 (-0.060–0.004)	0.083	-0.014 (-0.050–0.023)	0.453
SGLT-2 Inhibitors	0.178 (-0.227–0.583)	0.384	0.146 (-0.109–0.400)	0.259	0.054 (-0.221–0.329)	0.694	0.194 (0.081–0.307)	0.001	0.147 (0.057–0.236)	0.001	0.249 (-0.088–0.585)	0.146	0.275 (-0.131–0.680)	0.183
RAS Inhibitors	-0.007 (-0.395–0.380)	0.970	-0.081 (-0.326–0.163)	0.511	0.067 (-0.265–0.398)	0.690	-0.105 (-0.196–-0.013)	0.025	-0.017 (-0.094–0.060)	0.669	-0.054 (-0.315–0.208)	0.685	-0.110 (-0.449–0.228)	0.519
UACR	-0.343 (-0.670–-0.015)	0.041	-0.089 (-0.165–-0.014)	0.021	-0.019 (-0.138–0.100)	0.749	-0.104 (-0.135–-0.072)	<0.001	-0.020 (-0.048–0.008)	0.153	-0.109 (-0.188–-0.031)	0.007	-0.071 (-0.182–0.039)	0.204
BMI	-0.010 (-0.049–0.030)	0.625	0.014 (-0.008–0.035)	0.207	0.010 (-0.016–0.035)	0.447	0.004 (-0.008–0.016)	0.545	-0.003 (-0.013–0.007)	0.556	0.005 (-0.028–0.039)	0.754	0.004 (-0.034–0.042)	0.831
SBP	-0.002 (-0.011–0.007)	0.687	-0.008 (-0.016–-0.001)	0.034	-0.003 (-0.012–0.006)	0.510	-0.004 (-0.007–-0.001)	0.004	-0.003 (-0.005–-0.001)	0.014	-0.008 (-0.015–0.000)	0.046	-0.002 (-0.010–0.006)	0.655
eGFR	-0.015 (-0.027–-0.004)	0.008	-0.001 (-0.023–0.021)	0.915	-0.004 (-0.030–0.021)	0.730	-0.003 (-0.009–0.004)	0.372	-0.033 (-0.038–-0.027)	<0.001	-0.014 (-0.026–-0.001)	0.035	-0.015 (-0.029–-0.001)	0.035
HbA1c	-0.105 (-0.223–0.014)	0.084	-0.041 (-0.107–0.026)	0.226	0.012 (-0.070–0.095)	0.765	-0.015 (-0.061–0.030)	0.504	-0.019 (-0.057–0.019)	0.322	-0.061 (-0.201–0.080)	0.397	-0.127 (-0.307–0.054)	0.168

In the subgroup analysis of the UACR and eGFR categories by multiple-adjusted model (Model 2), SD (UACR) was significantly associated with the three-year eGFR slope in the eGFR 60–90 mL/min/1.73 m^2^ groups. SD (eGFR) was significantly associated with the three-year eGFR slope in all categories except the eGFR < 60 mL/min/1.73 m^2^ group. SD (HbA1c) and SD (SBP) were not significantly associated with the three-year eGFR slope in the multiple-adjusted model. After sensitivity analysis, similar associations were found in all participants in the non-medication changes group by multiple-adjusted model (Model 2), except for baseline SBP (Supplementary Table 1).

## Discussion

This study aimed to evaluate the seasonal variability of UACR and eGFR and associate any variability with the three-year eGFR slope in Japanese with T2D, independent of previously reported factors. This study indicated two major findings. First, the UACR in persons with T2D exhibited seasonal variability, with a decrease in spring to autumn and an increase in winter, while eGFR showed no seasonal variability. Second, variabilities in UACR and eGFR were associated with a three-year eGFR slope in Japanese with T2D, independent of known factors (such as SGLT-2 inhibitors) as well as the baseline eGFR and blood pressure.

### The seasonal variability of UACR

In previous studies evaluating the variability of UACR or urinary albumin excretion (UAE), visit-to-visit variabilities in UACR and UAE were assessed at random points, independent of season [27–31]. In these studies, the median coefficient of variation (CV) of UACR was reported to be approximately 30 to 80%, with CV values increasing as the observation period increased (e.g., 32.5% in a three-to six-month study or 67.7 to 81.6% in a 7.9-year study) [28–30]. In our study, the CV of UACR in all participants was 86 (63–105) %, similar to previous results. To our best knowledge, only one report evaluated seasonal variability and that one-year observational study assessing UACR variability in Japanese with T2D and early diabetic nephropathy reported higher winter versus summer values [16]. Our results are comparable and add that seasonal UACR variability manifests during any stages of diabetic nephropathy (microalbuminuria, plus normoalbuminuria and macroalbuminuria), since all stages were included.

We assumed that UACR variability was affected by other seasonally variable factors, such as SBP, blood glucose, and dietary habits, as some fluctuations are associated with temperature changes [32, 33]. A large, multicenter study evaluating seasonal variations in Japanese with T2D reported lower SBP and HbA1c achievement rates in winter compared with summer [34]. This observed variability was associated with albuminuria pathogenesis in persons with T2D [35, 36]. Moreover, seasonal fluctuations in lifestyle activities (physical activity and eating behaviors) [37] and increased salt [38] or protein [39] intake during winter might have also affected UACR variability.

### Associations between the three-year eGFR slope and the variability of UACR and eGFR

Although few studies evaluating relationships between UACR variability and renal function exist, Lin et al. reported associations between visit-to-visit UACR variability and long-term deterioration of renal function in persons with T2D and early-stage DKD (eGFR ≥ 60 mL/min/1.73 m^2^ and UACR < 300 mg/g) in a single-center, retrospective, observational study [27]. There, they used the albuminuria variability score (AVS) to represent UACR variability, which was defined as the number of changes in UACR above 3.39 mg/mmol (30 mg/g). Consistent with our results, they reported that higher AVS was negatively associated with average annual change of eGFR (independent of age), baseline eGFR/UACR, and hypertension. Here, we extend these observations with five new points. First, we adjusted for SGLT-2 inhibitors as independent variables due to known renoprotective effects [40]. Second, we adjusted both the baseline eGFR and its variability (SD [eGFR]) for independent variables, since eGFR variability was previously associated with eGFR slope in advanced CKD [24] or linked to cardiovascular outcomes [41]. Third, we used SD (UACR) as a continuous variable, calculating it using values measured three times a year in fixed seasons to obtain both long-term and seasonal variabilities of UACR, unlike the Lin et al. report that used randomly measured UACR values from ten observation years. Fourth, we enrolled a wider range of participants, including patients with normo- to macroalbuminuria and normal to eGFR < 60 mL/min/1.73 m^2^, but Lin et al. enrolled patients with UACR < 300 mg/g and eGFR ≥ 60 mL/min/1.73 m^2^. Consequently, in our study, SD (UACR) was significantly associated with eGFR slope only in the group of eGFR > 90 mL/min/1.73 m^2^ (all study participants group) or eGFR ≥ 60 mL/min/1.73 m^2^ (non-medication changes group), whose result was comparable to the result of Lin et al. Fifth, we performed a sensitivity analysis in the non-medication-changes group to exclude medication change effects on UACR and eGFR.

The SD (eGFR), similar to the SD (UACR), was significantly and independently associated with the three-year eGFR slope. Furthermore, when examining UACR and eGFR categories, a significant association was observed in a broader population compared to the SD (UACR). This is consistent with previous reports indicating renal outcome links to eGFR variability in persons with T2D [14, 15], differing in only two aspects. First, those previous outcomes were the worsening of nephropathy, including new-onset of macroalbuminuria, end-stage kidney disease, renal death, and doubling of creatinine [14], as well as the duration to dialysis or dialysis-free death [15]. In contrast, we used eGFR slope to represent renal outcomes, serving as a surrogate marker for the composite renal endpoint (*i.e.*, doubling of serum creatinine, progression to end-stage renal failure, and renal insufficiency with renal replacement therapy) [42]. Second, previous studies focused on normo-to-microalbuminuria [14], or were limited to CKD stage 3–4 [15], whereas we evaluated broader populations for both UACR and eGFR categories. Thus, our findings provide crucial evidence for the management and monitoring of DKD in persons with T2D.

In our study, associations between baseline HbA1c level/SD (HbA1c) and the three-year eGFR slope were insignificant. The U.K. Prospective Diabetes Study (UKPDS) showed that intensive glycemic control reduces the development of microalbuminuria, proteinuria, and two-fold creatinine increase [43]. However, significant risk reduction was only observed after more than nine years in the surrogate endpoints of microalbuminuria and two-fold creatinine increase [43]. A meta-analysis evaluating the association between HbA1c variability and renal disease included studies longer than our observation period (longest median observation: 15.9 years) [11]. Therefore, a short observation period may have precipitated our failure to associate renal function changes with observed baseline HbA1c level and SD (HbA1c) values.

The baseline UACR was also insignificant in our study. Since there was a significant association in the group with a UACR ≥ 300 mg/g, we considered those with classic diabetic nephropathy at baseline to have demonstrated an association consistent with previous reports [7]. On the other hand, we considered the possibility that our cohort included non-albuminuric renal dysfunction patients, characterized by low eGFR and normal albuminuria. A prospective observational study reported a prevalence of 56.6% for people with eGFR < 60 ml/min/1.73 m^2^ and normoalbuminuria [44]. The presence of individuals with these characteristics may have influenced the results of the multivariate analysis.

The UKPDS reported baseline urinary albumin and serum creatinine levels as independently associated with renal function changes at 15 years [45]. Our results suggest that UACR and eGFR variabilities are also independently associated with renal function changes, in addition to baseline eGFR levels. While the mechanism by which UACR variability relates to eGFR slope remains unelucidated, we posit that blood pressure variability is a confounding factor for UACR variability and eGFR slope change since it is associated with CKD progression [46] or the worsening of albuminuria [47] in persons with T2D. However, SBP variability was not significantly associated with the three-year eGFR slope in our study. This may be due to inherent discrepancies between “office” and “home” blood pressure readings [48].

Hydration status might also contribute to UACR variability. Serum osmolality and urine specific gravity vary by season, with urine reported as more concentrated during winter [49]. Vasopressin has also been reported as a seasonal factor, since its secretion is stimulated by increased plasma osmolality [50], decreased blood volume [50], and increased protein intake [51]. Vasopressin or copeptin (a surrogate marker for vasopressin) are reported to be associated with increased urinary albumin excretion [52, 53] or decreased eGFR [54] and also show seasonal variation, being higher in winter and lower in summer [55]. These collective findings indicate that, during winter, seasonal increases in vasopressin may result in increased urinary albumin excretion.

Associations between SD (eGFR) and the eGFR slope may be attributed to repeated, transient renal impairment, leading to potential renal deterioration. Factors influencing renal impairment include severe illnesses, surgeries, the use of nephrotoxic drugs (antibiotics, non-steroidal anti-inflammatory drugs [NSAIDs], and contrast agents), and dehydration [56], all of which are common in routine diabetes care. Although these exposure factors were not incorporated into the multivariable analysis model due to the retrospective nature of the current study, they may have been confounding factors.

### Strengths and limitations

Our study has two strengths. First, we reduced selection bias by complementing missing cases with multiple imputation methods rather than complete data analysis. Second, we performed a multivariable analysis on factors associated with eGFR slope by adding a wide range of known, associated factors along with SD (UACR) and SD (eGFR) as independent variables to minimize the effect of confounding factors.

We acknowledge four main limitations. First, as this single-center study involved only a single racial group (Japanese), larger cohort multicenter and multi-ethnic studies are required for generalization of results. Second, although the SD (UACR) and SD (eGFR) were used as variability indices, these may have reflected not only fluctuation but also the amplitude of change over the 3 years. Leong et al. reported that patterns with a progressive UACR increase are considered to have a higher coefficient of variation (CV) than those with a constant range of fluctuation [29]. Although the progressive group in that study increased two- to three-fold or more from baseline, in our study, UACR changed only 1.3-fold over 3 years, from a mean of 124 mg/g to 165 mg/g (Fig. 2). This observation implies that the amplitude of the UACR change had no significant impact on SD (UACR). Moreover, SD (eGFR) was previously validated as a variability index [15]. Third, although we used multiple regression analysis while assuming a linear change in eGFR, these changes are often nonlinear [9], so our results should be carefully extrapolated for clinical practice. Fourth, we could not fully adjust for confounding factors affecting the eGFR slope due to the retrospective study nature. Further prospective studies should add factors (*e.g.*, vasopressin, salt intake, protein intake, fluid intake, and nephrotoxic drugs); such results may help elucidate the pathogenesis of UACR variability as related to the eGFR slope.

## Conclusions

The present study demonstrates both the seasonal variability of UACR and that UACR and eGFR variabilities are associated with a three-year eGFR slope in persons with type 2 diabetes, independent of the baseline eGFR and blood pressure. When evaluating UACR and eGFR in persons with T2D, seasonal variability in UACR becomes an important consideration which may modulate the long-term eGFR slope and variability.

### Supplementary Information

Supplementary Material 1

## Data Availability

The data that support the findings of this study are not openly available due to reasons of sensitivity and are available from the corresponding author upon reasonable request.

## Abbreviations

ADVANCE: Action in Diabetes and Vascular Disease: PreterAx and DiamicroN-MR Controlled Evaluation
AVS: Albuminuria variability score
BMI: Body mass index
BW: Body weight
CKD: Chronic kidney disease
DBP: Diastolic blood pressure
DKD: Diabetic kidney disease
DPP-4: Dipeptidyl peptidase-4
eGFR: Estimated glomerular filtration rate
GLP-1RAs: Glucagon-like peptide-1 receptor agonists
HDL-C: High-density lipoprotein cholesterol
HPLC: High-performance liquid chromatography
LDL-C: Low-density lipoprotein cholesterol
NSAIDs: Non-steroidal anti-inflammatory drugs
RAS: Renin-angiotensin system
SBP: Systolic blood pressure
SD: Standard deviation
SGLT-2: Sodium-glucose cotransporter-2
T2D: Type 2 diabetes
TG: Triglyceride
UA: Uric acid
UACR: Urinary albumin to creatinine ratio
UAE: Urinary albumin excretion
