# Supplementary material for: Variability of urinary albumin to creatinine ratio and eGFR are independently associated with eGFR slope in Japanese with type 2 diabetes: a three-year, single-center, retrospective cohort study
Source: BMC Nephrol. 2024 Aug 16;25:264. doi: 10.1186/s12882-024-03699-4 (PMC11330002; doi:10.1186/s12882-024-03699-4)
Supplement: Supplementary file 1 — Supplementary Material 1 [file 12882_2024_3699_MOESM1_ESM.docx]

**Supplementary Table 1.** Associations between three-year change in eGFR slope and variability of UACR and eGFR (non-medication-changes group)

| Independent Variables | All |  | UACR Category (mg/g) |  |  |  |  |  | eGFR Category (mL/min/1.73 m^2^) |  |  |  |  |  |
| --- | --- | --- | --- | --- | --- | --- | --- | --- | --- | --- | --- | --- | --- | --- |
|  |  |  | < 30 |  | 30–300 |  | 300 ≤ |  | 90 ≤ |  | 60–90 |  | < 60 |  |
|  | (n = 801) |  | (n = 428) |  | (n = 294) |  | (n = 79) |  | (n = 64) |  | (n = 412) |  | (n = 125) |  |
|  | β (95% CI) | P | β (95% CI) | P | β (95% CI) | P | β (95% CI) | P | β (95% CI) | P | β (95% CI) | P | β (95% CI) | P |
| SD (UACR) | -0.001 (-0.001–0.000) | <0.001 | -0.001 (-0.001–0.000) | 0.124 | -0.001 (-0.001–0.000) | 0.114 | -0.001 (-0.001–0.000) | 0.144 | 0.000 (-0.001–0.001) | 0.334 | -0.001 (-0.001–0.000) | <0.001 | -0.001 (-0.002–0.001) | 0.230 |
| SD (eGFR) | -0.168 (-0.189–-0.148) | <0.001 | -0.132 (-0.164–-0.099) | <0.001 | -0.185 (-0.227–-0.144) | <0.001 | -0.249 (-0.322–-0.175) | <0.001 | -0.179 (-0.269–-0.088) | <0.001 | -0.197 (-0.219–-0.176) | <0.001 | -0.113 (-0.197–-0.029) | 0.009 |
| SD (HbA1c) | 0.105 (-0.034–0.243) | 0.138 | 0.118 (-0.057–0.293) | 0.184 | 0.091 (-0.176–0.359) | 0.501 | 0.095 (-0.555–0.745) | 0.771 | -0.147 (-0.431–0.137) | 0.301 | 0.122 (-0.011–0.255) | 0.072 | 0.120 (-0.703–0.943) | 0.772 |
| SD (SBP) | 0.004 (-0.006–0.014) | 0.453 | -0.002 (-0.015–0.010) | 0.699 | 0.012 (-0.009–0.033) | 0.279 | 0.014 (-0.034–0.063) | 0.547 | -0.007 (-0.035–0.021) | 0.617 | 0.008 (-0.003–0.018) | 0.141 | -0.015 (-0.056–0.027) | 0.487 |
| SGLT-2 Inhibitors | 0.180 (0.080–0.280) | <0.001 | 0.126 (0.004–0.248) | 0.043 | 0.254 (0.070–0.438) | 0.007 | 0.204 (-0.233–0.642) | 0.352 | 0.190 (-0.021–0.400) | 0.076 | 0.147 (0.048–0.247) | 0.004 | 0.314 (-0.188–0.817) | 0.217 |
| RAS Inhibitors | -0.073 (-0.159–0.013) | 0.098 | -0.105 (-0.211–0.002) | 0.054 | -0.112 (-0.274–0.049) | 0.171 | 0.014 (-0.418–0.446) | 0.948 | 0.090 (-0.145–0.325) | 0.443 | -0.091 (-0.174–-0.007) | 0.034 | 0.003 (-0.403–0.409) | 0.989 |
| UACR | -0.015 (-0.046–0.016) | 0.355 | -0.031 (-0.100–0.039) | 0.387 | 0.021 (-0.104–0.146) | 0.739 | -0.046 (-0.501–0.408) | 0.837 | -0.029 (-0.120–0.062) | 0.520 | -0.015 (-0.044–0.014) | 0.317 | 0.007 (-0.138–0.152) | 0.924 |
| BMI | 0.002 (-0.008–0.012) | 0.705 | 0.003 (-0.010–0.015) | 0.680 | 0.000 (-0.023–0.022) | 0.976 | -0.011 (-0.058–0.036) | 0.649 | 0.004 (-0.015–0.024) | 0.667 | -0.002 (-0.013–0.008) | 0.685 | 0.007 (-0.040–0.054) | 0.770 |
| SBP | -0.002 (-0.004–0.000) | 0.068 | -0.001 (-0.004–0.002) | 0.541 | -0.003 (-0.008–0.001) | 0.162 | -0.003 (-0.013–0.008) | 0.604 | -0.001 (-0.008–0.006) | 0.687 | -0.001 (-0.004–0.001) | 0.204 | -0.008 (-0.018–0.003) | 0.147 |
| eGFR | -0.019 (-0.023–-0.015) | <0.001 | -0.018 (-0.024–-0.012) | <0.001 | -0.023 (-0.032–-0.015) | <0.001 | -0.016 (-0.029–-0.003) | 0.019 | -0.012 (-0.032–0.008) | 0.234 | -0.031 (-0.037–-0.024) | <0.001 | -0.009 (-0.029–0.010) | 0.346 |
| HbA1c | -0.025 (-0.067–0.017) | 0.243 | 0.027 (-0.030–0.085) | 0.346 | -0.047 (-0.120–0.027) | 0.211 | -0.128 (-0.287–0.030) | 0.110 | 0.047 (-0.024–0.117) | 0.189 | -0.020 (-0.063–0.023) | 0.361 | -0.154 (-0.375–0.067) | 0.169 |

Data are expressed as the estimated partial regression coefficient (β) (95% confidence interval [95% CI]). Baseline UACR was used after log transformation. In this multiple linear regression analysis, we adjusted by age, sex, smoking, duration of diabetes, past medical history or comorbidity (hypertension, dyslipidemia, diabetic retinopathy, and/or cardiovascular disease), and therapeutic agents (SGLT-2 inhibitors, RAS inhibitors, GLP-1 receptor agonists, calcium channel blockers, mineralocorticoid receptor antagonist, loop diuretics, and/or thiazides), baseline data (UACR, BMI, SBP, eGFR, and HbA1c), and SD of UACR, eGFR, HbA1c, and SBP. *UACR* urinary albumin to creatinine ratio, *SD* standard deviation, *eGFR* estimated glomerular filtration rate, *SBP* systolic blood pressure, *SGLT-2* sodium-glucose cotransporter-2, *RAS* renin-angiotensin system, *BMI* body mass index.
